# Pediatric falls ages 0–4: understanding demographics, mechanisms, and injury severities

**DOI:** 10.1186/s40621-018-0147-x

**Published:** 2018-04-10

**Authors:** Sofia Chaudhary, Janet Figueroa, Salah Shaikh, Elizabeth Williams Mays, Rana Bayakly, Mahwish Javed, Matthew Lee Smith, Tim P. Moran, Jonathan Rupp, Sharon Nieb

**Affiliations:** 1 Division of Emergency Medicine, Children’s Hospital of Philadelphia, Philadelphia, PA USA; 2 Department of Pediatrics, Emory University School of Medicine, Atlanta, GA USA; 3 Rollins School of Public Health, Emory University, Atlanta, GA USA; 4 Department of Trauma and Surgical Services, Grady Health System, Atlanta, GA USA; 5 Georgia Department of Public Health, Chronic Disease, Healthy Behaviors and Injury Epidemiology Section, Atlanta, GA USA; 6 Safe Kids GA, Children’s Healthcare of Atlanta, Atlanta, GA USA; 7 Center for Population Health and Aging, Texas A&M University, College Station, TX USA; 8 Department of Environmental and Occupational Health, Texas A&M School of Public Health, College Station, TX USA; 9 Department of Health Promotion and Behavior, College of Public Health, The University of Georgia, Athens, GA USA; 10 Department of Emergency Medicine, Emory University, Atlanta, GA USA

**Keywords:** Falls, Unintentional, Pediatrics

## Abstract

**Background:**

Pediatric unintentional falls are the leading cause of injury-related emergency visits for children < 5 years old. The purpose of this study was to identify population characteristics, injury mechanisms, and injury severities and patterns among children < 5 years to better inform age-appropriate falls prevention strategies.

**Methods:**

This retrospective database study used trauma registry data from the lead pediatric trauma system in Georgia. Data were analyzed for all patients < 5 years with an international classification of disease, 9th revision, clinical modification (ICD-9 CM) external cause of injury code (E-code) for unintentional falls between 1/1/2013 and 12/31/2015. Age (months) was compared across categories of demographic variables, injury mechanisms, and emergency department (ED) disposition using Kruskal-Wallis ANOVA and the Mann Whitney U test. The relationships between demographic variables, mechanism of injury (MOI), and Injury Severity Score (ISS) were evaluated using multinomial logistic regression.

**Results:**

Inclusion criteria were met by 1086 patients (median age = 28 months; 59.7% male; 53.8% White; 49.1% <  1 m fall height). Younger children, < 1-year-old, primarily fell from caregiver’s arms, bed, or furniture, while older children sustained more falls from furniture and playgrounds. Children who fell from playground equipment were older (median = 49 months, *p* < 0.01) than those who fell from the bed (median = 10 months), stairs (median = 18 months), or furniture (median = 19 months). Children < 1 year had the highest proportion of head injuries including skull fracture (63.1%) and intracranial hemorrhage (65.5%), 2-year-old children had the highest proportion of femur fractures (32.9%), and 4-year-old children had the highest proportion of humerus fractures (41.0%). Medicaid patients were younger (median = 24.5 months, p < 0.01) than private payer (median = 34 months). Black patients were younger (median = 20.5 months, *p* < 0.001) than White patients (median = 29 months). Results from multinomial logistic regression models suggest that as age increases, odds of a severe ISS (16–25) decreased (OR = 0.95, CI = 0.93–0.97).

**Conclusions:**

Pediatric unintentional falls are a significant burden of injury for children < 5 years. Future work will use these risk and injury profiles to inform current safety recommendations and develop evidence-based interventions for parents/caregivers and pediatric providers.

**Electronic supplementary material:**

The online version of this article (10.1186/s40621-018-0147-x) contains supplementary material, which is available to authorized users.

## Background

Unintentional falls were the leading cause of nonfatal injury among children 0–4 years old from 2000 to 2015 in the United States (Centers for Disease Control and Prevention, 2017a). Over the past decades, both national and local childhood injury prevention efforts have provided education and interventions in an effort to reduce these injuries. Amongst the successful local programs were the “Children Can’t Fly” and “Kids Can’t Fly” campaigns in New York City and Boston, respectively. Within the first 10 years of implementation, these public health campaigns resulted in up to a 96% reduction of window falls for children < 5-years-old (Harris et al., 2011). Nationally, the American Academy of Pediatrics (AAP) has been at the forefront of providing pediatric caregiver and community education as well as fall prevention strategies through Council on Injury, Violence, and Poison Prevention (COIVPP) policy statements about injuries associated with infant walkers, shopping carts, trampolines, and falls from heights (American Academy of Pediatrics, 2001a; American Academy of Pediatrics, 2006; American Academy of Pediatrics, 2012; American Academy of Pediatrics, 2001b). Despite these injury prevention efforts, unintentional pediatric falls have remained a significant cause of injury, medical morbidity, and cost to the healthcare system in the youngest population. According to the Centers for Disease Control and Prevention, in 2010, unintentional falls in children < 5 years led to 1,077,652 emergency department (ED) visits with lifetime medical costs of over 2.5 billion dollars as well as 22,451 hospitalizations with lifetime medical costs of over 750 million dollars (Centers for Disease Control and Prevention, 2017b). These data exemplify the magnitude of the financial and medical burdens caused by pediatric fall-related injuries.

Prior studies on pediatric falls have evaluated specific mechanisms of falls (windows, stairs, furniture) (Harris et al., 2011; Pressley & Barlow, 2005; Zielinski et al., 2012; Pomerantz et al., 2012; Kendrick et al., 2015; Kendrick et al., 2016) or specific injuries sustained (head injury) (Love et al., 2009; Ibrahim et al., 2012). Few population-based studies have examined overall risk factors and injury mechanisms for falls as a function of age (Khambalia et al., 2006; Pitone & Attia, 2006; Unni et al., 2012; Wang et al., 2013). Fall injury prevention efforts are enhanced when population risk factors, typical injury mechanisms, and injury patterns according to developmental age are used to provide targeted recommendations. Better understanding of expected injury patterns from fall mechanisms can guide clinicians to distinguish between child abuse and unintentional injuries (Thompson et al., 2013).

The primary objectives of our study were to examine population characteristics, mechanisms of injury (MOI), injury patterns, and injury severities from falls among children < 5-years-old. The secondary objectives were to identify trends and patterns from these data to make recommendations for age-directed fall prevention education and interventions.

## Methods

A retrospective analysis was performed on trauma registry data for children ages 0–4 years presenting to Children’s Healthcare of Atlanta (CHOA) with a fall-related injury between January 1, 2013 and December 31, 2015. CHOA, the lead pediatric referral center for the state of Georgia, includes two tertiary free-standing pediatric trauma hospitals: level 1 (regional) and level 2 (suburban). Data extraction was based on chief complaint of fall injury and international classification of disease, 9th revision, clinical modification (ICD-9 CM) unintentional fall-related external cause of injury codes (E-codes) (Additional file 1). Patients were excluded if they showed evidence of child abuse (i.e., by diagnoses code or by documentation of diagnosis of child abuse or high suspicion of child abuse by child advocacy team in the medical record). Human studies approval was granted by the CHOA Institutional Review Board.

CHOA trauma registry inclusion criteria during the study period followed National Trauma Data Standard Dataset (NTDSD) standards, which indicated all patients: 1) Sustained a traumatic injury and had at least one of the following injury ICD-9 CM diagnosis codes (800.00–959.9); 2) Be either admitted for 23 h or transferred to/from another facility (regardless of length of stay [LOS] or discharge from ED), died (regardless of LOS), admitted to the ICU (regardless of LOS), dead on arrival, or have an unscheduled readmission associated with trauma within 72 h of discharge from the first visit. Patients evaluated in the ED, not transferred from another institution, and discharged home are not included in the trauma registry. Exclusion criteria for NTDSD included having the following ICD-9 CM diagnosis codes: 905–909 (late effects of injury), 910–924 (blisters, contusions, abrasions, and insect bites), and 930–939 (foreign bodies). CHOA trauma registry data contained 70% of the state pediatric falls injury-related patients included in the Georgia Central Trauma Registry Database (GCTR) for the same age group during our study period. GCTR is a statewide trauma database that follows NTDSD inclusion criteria and collects trauma registry data from all level (I through IV) trauma centers across the state.

A standard dataset was extracted from the CHOA trauma registry including demographic variables (age, gender, race, ethnicity, payer, street address, and zip code) and non-demographic variables (medical record number [MRN], height of fall, MOI, injuries sustained, procedures performed, Injury Severity Score [ISS], and ED disposition). MOI, injuries sustained, and procedures performed were extracted from ICD-9 CM E-codes included in the trauma registry. Payer (Medicaid, private, or other primary insurance) was selected as proxy for socioeconomic status (SES). Fall injury mechanisms were differentiated using the following distinctions: falls on the same level versus multilevel falls or falls from stair/steps, falls from household furniture versus falls from stairs/steps or playground, and low versus high height falls. These distinctions were made to identify age-associated risk factors, injury trends, and provide targeted falls prevention recommendations. Thus, MOI was subcategorized into general MOI (fall on same level, fall from stairs/steps, multilevel fall, or other) and specific MOI (fall from bed, furniture, stairs, playground, or other) through coding by keyword search from the initial MOI ICD-9-CM E-codes (Additional file 1) pulled from the trauma registry. Medical records were individually reviewed to investigate circumstances around falls and general MOI were further characterized by age according to narratives and by product coding (Additional file 1). Height of fall was grouped into 4 categories (< 1 m [< 3.3 ft], 1 m-6 m [3.3 ft–19.6 ft], > 6 m [> 19.6 ft], and unknown height) ranging from low-level fall to high-level fall. The Injury Severity Score (ISS) is an established scoring system used to calculate trauma severity by trauma services and is based on the sum of the squares of the highest Abbreviated Injury Score (AIS) for the three most severely injured body regions (Baker et al., 1974). Severity of injury was trichotomized based on standard ISS categories: mild (1–8), moderate (9–15), moderate/severe (16–25), and severe/critical (25+).

All statistical analyses were performed on de-identified data using SAS 9.4 (Cary, NC) and SPSS (v. 24; Armonk, NY). Descriptive statistics including medians and counts/frequencies were reported for variables of interest (listed above). Categorical variables were compared using the chi-square or Fisher’s exact tests. Continuous variables were compared using Mann-Whitney U tests for two-level variables and Kruskal-Wallis ANOVA for three or more level variables. Post-hoc comparisons using adjusted standardized residuals (z) and Bonferroni correction were conducted if omnibus *p*-values were significant (i.e. *p* < 0.05) in order to determine the sources of significant differences between variables with 3+ response categories (Agresti, 2007; Garcia-Perez & Nunez-Anton, 2003; Beasley & Schumacker, 1995). To identify potential age-specific high-risk populations, age was assessed as a continuous variable (in months). Results were reported as medians due to non-normal distributions. A multinomial logistic regression model was used to predict the odds of a moderate or severe ISS score relative to a mild score (odds ratios (OR) and 95% confidence intervals (CI) are presented). Multinomial logistic regression was used because the proportional odds assumption for ordinal regression was not met. Variables included in the multivariate model were age, gender, race, SES, and MOI.

## Results

During the three-year period, 1086 patients 0–4 years old with a fall-related injury were included in the CHOA trauma registry and met study criteria. The majority of patients (*n* = 606, 55.8%) presented to the level 2 (suburban) pediatric trauma center and 44.2% (*n* = 480) presented to the level 1 (regional) pediatric trauma center. There were no fatalities.

### Demographics

Tables 1 and 2 present sample demographics by age. The majority of falls patients were male (*n* = 648, 59.7%), White (*n* = 584, 53.8%), non-Hispanic (*n* = 944, 86.9%), and on Medicaid insurance (*n* = 718, 66.1%). The median age of patients was 28 months. Black patients were significantly younger than White patients (median = 20.5 months vs. median = 29 months). Medicaid patients were significantly younger than private payer patients (median = 24.5 months vs. median = 34 months). In post-hoc analyses (tables not shown), Black patients were less likely to be on private insurance than Medicaid (14.6% vs. 38.3%, z = − 6.7 vs. + 6.3, respectively). Conversely, White patients were more likely to be on private insurance than on Medicaid (77.5% vs. 46.1%, z = + 8.8 vs. -8.5, respectively). For comparisons by primary payer and comparisons by race there were no significant differences in gender, mechanisms of injury, or ISS level.

**Table 1:** Demographics for falls population in years, ages 0–4 (2013–2015)

Demographics	All patients (*N* = 1086)	Age < 1 (*N* = 326)	1 year (*N* = 150)	2 year (*N* = 209)	3 year (*N* = 181)	4 year (*N* = 220)
Gender						
Male n(%)	648 (59.7%)	189 (58.0%)	94 (62.7%)	132 (63.2%)	109 (60.2%)	124 (56.4%)
Female n(%)	438 (40.3%)	137 (42.0%)	56 (37.3%)	77 (36.8%)	72 (39.8%)	96 (43.6%)
Race						
White n(%)	584 (53.8%)	156 (47.9%)	77 (51.3%)	116 (55.5%)	103 (56.9%)	132 (60.0%)
Black n(%)	334 (30.8%)	130 (39.9%)	47 (31.3%)	63 (30.1%)	47 (26.0%)	47 (21.4%)
Other n(%)	168 (15.5%)	40 (12.3%)	26 (17.3%)	30 (14.4%)	31 (17.1%)	41 (18.6%)
Ethnicity						
Hispanic n(%)	142 (13.1%)	32 (9.8%)	26 (17.3%)	25 (12.0%)	26 (14.4%)	34 (15.5%)
Non-Hispanic n(%)	944 (86.9%)	295 (90.5%)	124 (82.7%)	184 (88.0%)	155 (85.6%)	186 (84.5%)
Primary Payer						
Medicaid n(%)	718 (66.1%)	246 (75.5%)	107 (71.3%)	132 (63.2%)	110 (60.8%)	123 (55.9%)
Private n(%)	269 (24.8%)	69 (21.2%)	24 (16.0%)	53 (25.4%)	46 (25.4%)	77 (35.0%)
Other n(%)	99 (9.1%)	11 (3.4%)	19 (12.7%)	24 (11.5%)	25 (13.8%)	20 (9.1%)

**Table 2:** Age (months) by demographics, disposition, MOI, and ISS

Predictor	% of Total sample	Median Age (M)	SIQR	p*^,a^
Sex				.69
Male	59.7	27.0	17.0	
Female	40.3	28.0	18.0	
Payer				<.001
Medicaid	66.1	24.5	16.5	
Private	24.8	34.0	19.5	
Other	9.1	32.0	13.0	
Race				<.001
Black	30.9	20.5	16.0	
White	55.0	29.0	18.0	
Other	14.1	29.0	18.0	
Ethnicity				.06
Hispanic	13.1	28.0	17.0	
Non-Hispanic	86.7	27.0	17.5	
General MOI				<.001
Fall on the Same Level	25.1	34.0	13.5	
Fall on/from Stairs	5.5	18.0	12.5	
Multilevel Fall	63.1	22.0	17.5	
Other	6.3	33.5	12.5	
Specific MOI (specific)				<.001
Bed	16.0	10.0	11.5	
Furniture	8.0	19.0	16.5	
Playground Equipment	9.1	49.0	8.0	
Stairs	5.6	18.0	12.5	
Other	61.2	29.0	17.0	
Height of fall				.35
Fall-Under 1 m (< 3.3 ft)	49.1	27.0	17.0	
Fall – 1 m - 6 m (3.3 ft–19.6 ft)	20.3	27.0	18.0	
Fall – Over 6 m (> 19.6 ft)	0.9	36.0	7.5	
Fall – Height unknown	29.7	27.0	18.0	
ISS Levels				<.001
1–8	63.3	32.0	18.5	
9–15	31.7	23.0	14.0	
16–25	5.1	9.0	10.5	
ED disposition				.01
Floor Bed	72.0	27.0	18.0	
Home without Services	17.5	29.5	16.0	
Intensive Care Unit	4.9	25.0	22.0	
Operating Room	5.6	34.0	13.0	

*Mann-Whitney U or Kruskal-Wallis Test

Note: SIQR: semi-interquartile range

^a^Posthoc comparisons shown in Additional file 1: Table S3

### Mechanisms of injury

#### Height of fall

The majority of the falls were from a low height of < 1 m (3.3 ft) (*n* = 533, 49.1%) (Table 2). There were 10 children (0.9%) that fell from a height > 6 m (> 19.6 ft); this included three children 1–2 years old (0.3%) who fell from balcony or building structures, six children 2–4 years old (0.6%) who fell from windows at least two stories high, and one 4-year-old child (0.1%) who fell from a playground surface (trampoline).

#### General and specific injury mechanisms

As seen in Table 2, most patients experienced a multi-level fall (*n* = 685, 63.1%). Those who fell from multi-level (median = 22 months) or stairs (median = 18 months) were significantly younger than those who fell from the same level (median = 34 months). For multi-level falls, most children < 1-year-old fell from a caregiver’s arms or furniture; most children 1–2 years old fell from furniture; and most children 3–4 years old fell from furniture, outdoor surfaces, and playground surfaces. For same level falls, most children < 1-year-old fell from a caregiver’s arms; and most children 1–4 years old fell from running, slipping, or tripping. When looking at specific mechanisms of injury, those who fell from playground equipment (median = 49 months) were significantly older than those who fell from furniture (median = 19 months), stairs (media*n* = 18 months), or a bed (median = 10 months). Table 3 provides findings from medical records and product coding (Additional file 1) and shows a variety of fall mechanisms according to age.

**Table 3:** Specific mechanisms according to age in years

Category	Total (n)	< 1 year n(%)	1 year n(%)	2 years n(%)	3 years n(%)	4 years n(%)
Balcony	6	0	0	3 (50.0%)	3 (50.0%)	0 (0.0%)
Bed	177	88 (49.7%)	29 (16.4%)	26 (14.7%)	23 (13.0%)	11 (6.2%)
Bouncy house	6	0	0	2 (33.3%)	3 (50.0%)	1 (16.7%)
Bouncy seat	3	3 (100.0%)	0	0	0	0
Caregiver’s arms	156	131 (84.0%)	11 (7.1%)	8 (5.1%)	4 (2.6%)	2 (1.3%)
Chair	54	9 (16.7%)	13 (24.1%)	9 (16.7%)	9 (16.7%)	14 (25.9%)
Changing table	7	6 (85.7%)	0	1 (14.3%)	0	0
Couch	58	12 (20.7%)	9 (15.5%)	15 (25.9%)	13 (22.4%)	9 (15.5%)
Counter	27	21 (77.8%)	2 (7.4%)	2 (7.4%)	1 (3.7%)	1 (3.7%)
Crib	5	1 (20.0%)	4 (80.0%)	0	0	0
Deck	3	0	0	0	3 (100.0%)	0
Other furniture	10	2 (20.0%)	1 (10.0%)	1 (10.0%)	3 (30.0%)	3 (30.0%)
High chair	7	1 (14.3%)	5 (71.4%)	1 (14.3%)	0	0
Playground	105	1 (1.0%)	6 (5.7%)	14 (13.3%)	26 (24.8%)	58 (55.2%)
Porch	11	0 (0.0%)	2 (18.2%)	3 (27.3%)	2 (18.2%)	4 (36.4%)
Shopping cart	13	2 (15.4%)	2 (15.4%)	7 (53.8%)	1 (7.7%)	1 (7.7%)
Sports	9	0	2 (22.2%)	1 (11.1%)	1 (11.1%)	5 (55.6%)
Stairs	54	10 (18.5%)	11 (20.4%)	15 (27.8%)	10 (18.5%)	8 (14.8%)
Stroller	11	9 (81.8%)	0	2 (18.2%)	0	0
Table	21	11(52.4%)	3 (14.3%)	3 (14.3%)	3 (14.3%)	1 (4.8%)
Toy	7	0	0	3 (42.9%)	1 (14.2%)	3 (42.9%)
Trampoline	33	1 (3.0%)	4 (12.1%)	3 (9.1%)	7 (21.2%)	18 (54.5%)
Tree	3	0	0	0 (0.0%)	0	3 (100.0%)
Vehicle	13	1 (7.7%)	1 (7.7%)	4 (30.8%)	5 (38.5%)	2 (15.4%)
Wheeled toy	15	0	3 (20.0%)	3 (20.0%)	5 (33.3%)	4 (26.7%)
Window	23	0	3 (13.0%)	8 (34.8%)	8 (34.8%)	4 (17.4%)

#### Falls from caregiver’s arms

A majority of children who fell from caregiver’s arms (*n* = 156) were <  1-year-old (*n* = 131, 84.0%) (Table 3). Among the children who fell from caregiver’s arms, 17.9% (*n* = 28) were from another child (sibling, friend, or relative) holding the patient, 11.5% (n = 18) were while being carried on stairs, 10.9% (*n* = 17) were from being carried in an unbuckled car seat (using car seat as a carrier), 3.8% (*n* = 6) were from an adult caregiver falling asleep with the child (while on bed or other furniture), and 1.3% (n = 2) were from being carried in an unbuckled bouncy seat.

#### Falls from furniture

As seen in Table 3, falls from furniture were documented for patients 0–4 years old, but falls based on furniture type varied by age. Falls from beds (*n* = 177) were the most frequent mechanism of injury. Among the 27 children who fell from a counter, the majority were children < 1-year-old, and 59.3% (*n* = 16) resulted from placing baby products (car seat [4 unbuckled], bouncy seat [3 unbuckled], booster seat, or baby seat) on top of the counter. Falls from a table (*n* = 21) were predominantly among children < 1-year-old, three of which resulted from baby products (infant seat, car seat [unbuckled], and bathtub) being placed on the table.

#### Falls from playground and strollers

As seen in Table 3, the majority of children who fell from the playground were 4-year-olds (*n* = 58, 55.2%). Falls from playground (*n* = 105) included falls from monkey bars (*n* = 24, 22.9%), swings (*n* = 22, 21.0%), or slides (*n* = 15, 14.3%). Of the 11 children who fell from a stroller, the majority were < 1-year-old (*n* = 9, 81.8%). About half of stroller falls (*n* = 4, 44.4%) occurred because an unbuckled car seat was placed on top of the stroller or a stroller was being used on stairs.

### Injury severity scores

As seen in Tables 2, 63.3% (*n* = 687) of patients had a mild ISS, 31.7% (*n* = 344) had moderate ISS, and 5.1% (*n* = 55) had severe ISS. Table 4 presents findings from the multinomial logistic regression examining factors associated with ISS level (mild ISS served as the referent category). Older children were significantly less likely to have moderate (OR = 0.98, 95% CI = 0.97–0.98) or severe (OR = 0.95, 95% CI = 0.93–0.97) ISS. In the model comparing mild ISS to moderate ISS, females (OR = 0.63, 95% CI = 0.48–0.84), those who fell from multi-level (OR = 0.50, 95% CI = 0.35–0.69), and those who fell from stairs/steps (OR = 0.43, 95% CI = 0.22–0.84) were less likely to have a moderate ISS (relative to their respective referent categories). In the model comparing mild ISS to severe ISS, those who fell from 1 m-6 m (3.3 ft–19.6 ft) (OR = 2.45, 95% CI = 1.20–5.01) and those who fell from > 6 m (> 19.6 ft) (OR = 12.9, 95% CI = 1.80–93.29) were more likely to have a severe ISS (relative to those who fell from < 1 m [< 3.3 ft]).

**Table 4:** Multinomial logistic regression predicting ISS* level, N = 1086

Predictor	Odds Ratio	95% CI	*p*
ISS Level: 16–25 vs 1–8 (referent)			
Age (Months)	0.95	0.93; 0.97	<.001
Gender			
Male	Reference	Reference	
Female	0.94	0.52; 1.69	0.84
Race			
White	Reference	Reference	
Black	0.70	0.36; 1.38	0.31
Other	0.95	0.39; 2.33	0.91
Payer			
Medicaid	Reference	Reference	
Private	0.72	0.34; 1.56	0.41
Other Payer	0.80	0.23; 2.80	0.72
General MOI			
Same Level	Reference	Reference	
Multi-Level	0.97	0.37; 2.54	0.96
Stairs/Steps	0.70	0.15; 3.25	0.65
Other MOI	1.22	0.27; 5.52	0.80
Height of Fall			
< 1 m	Reference	Reference	
1 m–6 m	2.45	1.20; 5.01	0.01
> 6 m	12.9	1.80; 93.29	0.01
Unknown	0.83	0.38; 1.80	0.63
ISS Level: 9–15 vs 1–8 (referent)			
Age Months	0.98	0.97; 0.98	<.001
Gender			
Male	Reference	Reference	
Female	0.63	0.48; 0.84	0.002
Race			
White	Reference	Reference	
Black	1.18	0.86; 1.60	0.30
Other	0.66	0.42; 1.04	0.08
Payer			
Medicaid	Reference	Reference	
Private	1.17	0.84; 1.62	0.37
Other Payer	1.34	0.84; 2.15	0.22
General MOI			
Same Level	Reference	Reference	
Multi-Level	0.50	0.35; 0.69	<.001
Stairs/Steps	0.43	0.22; 0.84	0.01
Other MOI	0.58	0.31; 1.09	0.09
Height of Fall			
< 1 m	Reference	Reference	
1 m–6 m	1.3	0.89; 1.92	0.17
Over 6 m	1.1	0.20; 6.05	0.93
Unknown	1.07	0.78; 1.47	0.68

*ISS groups (1–8 = mild, 9–15 = moderate, 16–25 = severe)

### Procedures and injuries sustained

**Fig. 1:**
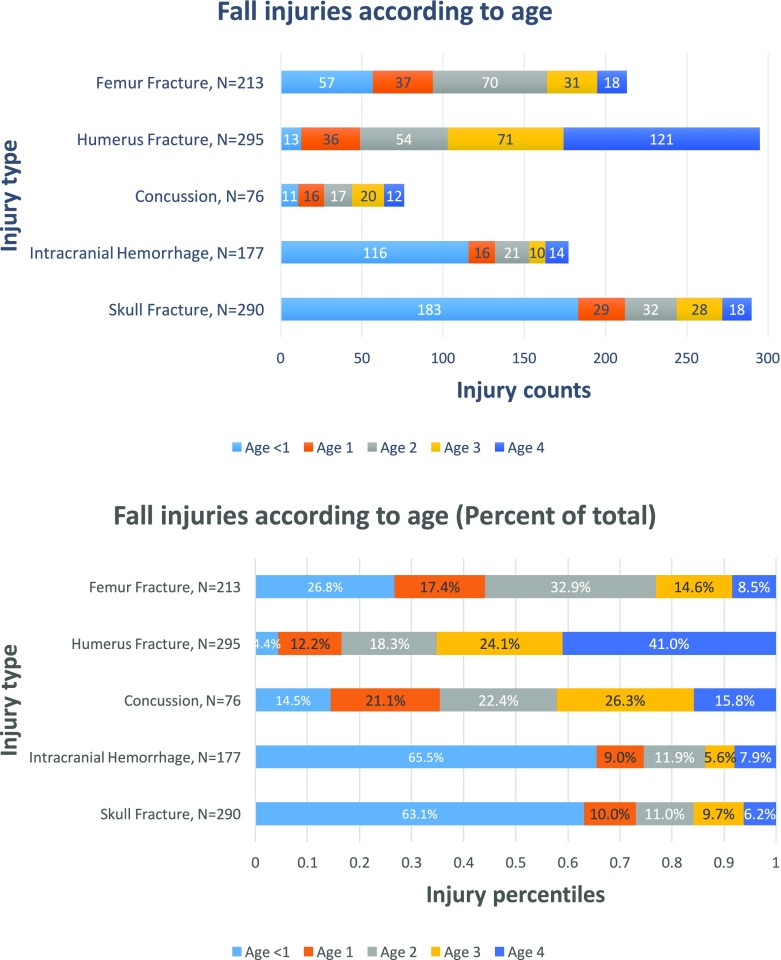
Fall injuries according to years of age

Table 5 presents medical procedures performed on pediatric fall patients. The highest percentage of neuroimaging (including MRI Brain, Head CT) (*n* = 336) and neurosurgical procedures (*n* = 20) were for children < 1-year-old (43.8% and 50.0%, respectively). In contrast, the highest percentage of orthopedic surgical procedures (including open and closed reduction with/without pinning) (*n* = 442) were seen among patients ages 2 years (23.8%), 3 years (24.0%), and 4 years (34.4%). This correlated with the number of particular injury patterns according to age (Fig. 1). Head injuries such as skull fractures (*n* = 290) and intracranial hemorrhage (*n* = 177) represented the majority of injuries for children < 1-year-old (63.1% and 65.5%, respectively). Two-year-old children had the highest percentage of femur fractures (32.9%), and 4-year-old children had the highest percentage of humerus fractures (41.0%).

**Table 5:** Procedures according to age

Procedure	Total	< Age 1 n(%)	Age 1 n(%)	Age 2 n(%)	Age 3 n(%)	Age 4 n(%)
Body CT	197	68 (34.5%)	28 (14.2%)	36 (18.3%)	32 (16.2%)	33 (16.8%)
Skeletal Series	180	126 (70.0%)	25 (13.9%)	22 (12.2%)	7 (3.9%)	0
Neuroimaging^a^	336	147 (43.8%)	45 (13.4%)	53 (15.8%)	48 (14.3%)	43 (12.8%)
Neurosurgical^b^	20	10 (50.0%)	3 (15.0%)	4 (20.0%)	3 (15.0%)	0
Orthopedic^c^	442	24 (5.4%)	55 (12.4%)	105 (23.8%)	106 (24.0%)	152 (34.4%)
Intubations	8	0	2 (25.0%)	4 (50.0%)	2 (25.0%)	0
Total Procedures	1183	375 (31.7%)	158 (13.4%)	224 (18.9%)	198 (16.7%)	228 (19.3%)

^a^Neuroimaging includes: Computerized axial tomography of head, magnetic resonance imaging of brain and brain stem, magnetic resonance imaging of spinal canal

^b^Neurosurgical procedures includes: craniotomy, ventriculostomy, elevation of skull fragments, incision of cerebral meninges

^c^Orthopedic procedures include: closed reduction of fracture without internal fixation, closed reduction of fracture with internal fixation, open reduction with internal fixation

Mechanisms for these injuries varied. Among children with a skull fracture (n = 290), 31.7% (*n* = 92) had fallen from a caregiver’s arms, 17.9% (*n* = 52) from a bed, 5.5% (*n* = 16) from stairs, 5.2% (*n* = 15) from a counter, 3.8% (*n* = 11) from a window, and 2.4% (*n* = 7) from a shopping cart. Among children with an intracranial hemorrhage (n = 177), 32.2% had fallen from caregiver’s arms (*n* = 57), 20.3% from a bed (*n* = 36), 6.8% from a counter (*n* = 12), 4.0% from stairs (n = 7), and 4.0% from a window (n = 7). Among the children with femur fractures (*n* = 213), 17.8% had fallen from a bed (*n* = 38), 13.6% from a caregiver’s arms (n = 29), 4.7% from stairs (*n* = 10), and 4.7% from a couch (n = 10). Among the children with a humerus fracture (*n* = 295), 21.7% had fallen from a playground (*n* = 64), 14.6% from a bed (*n* = 43), 8.1% from a couch (*n* = 24), 7.1% from a chair (n = 21), and 7.1% from a trampoline (n = 21).

### Disposition

Among patients admitted after their fall (*n* = 896, 82.5%), 87.2% (*n* = 781) were admitted to a general floor bed, 6.8% to the operating room (*n* = 61), and 6.0% to the intensive care unit (ICU) (*n* = 54). Those admitted to the operating room (median = 34 months) were significantly older than those admitted to the ICU (media*n* = 25 months) or floor bed (median = 27 months) (Table 2). Of all children included in the study, 69.1% (*n* = 750) were transferred from an outside facility to CHOA. Of the transfers admitted (*n* = 562), 5.9% were admitted to the ICU (*n* = 33) and 6.4% to the operating room (n = 36). Injuries from the 750 transferred patients included: skull fracture (*n* = 206, 27.5%), ICH (*n* = 119, 15.9%), concussion (*n* = 50, 6.7%), humerus fracture (*n* = 194, 25.9%), and femur fracture (*n* = 142, 18.9%). ISS for the 750 transferred patients were as follows: mild ISS (*n* = 475, 63.3%), moderate ISS (*n* = 238, 31.7%), and severe ISS (*n* = 37, 4.9%). Discharged patients (*n* = 190) included those with the following injuries: skull fracture (*n* = 44, 23.2%), ICH (*n* = 9, 4.7%), concussion (n = 25, 13.2%), humerus fracture (*n* = 28, 14.7%), and femur fracture (n = 3, 1.6%).

## Discussion

Pediatric falls are frequently seen among young children and can cause injuries requiring hospitalization. Our study illustrates how low-level falls can cause a variety of severe injuries and involve a variety of fall surfaces depending upon the child’s age. A prior systematic review found that young age, male sex, and low socioeconomic status were consistent risk factors for fall injuries among children ages 0–6 (Khambalia et al., 2006). Similarly, our study found that the majority of the children with falls were male and had Medicaid insurance. Although most of these falls were from a low height and with mild ISS, many sustained injuries requiring medical procedures. Younger children were found to have the most severe ISS and the most critical fall-related injuries were seen in children < 1-year-old. Children < 1-year-old also required the most imaging (neuroimaging, skeletal series, and body CT) and neurosurgical procedures given their injury patterns.

As children age, their mobility increases from generally being able to roll over at 4–5 months, sit up at 6 months, pull to a standing position at 9 months, walk starting at 12 months, and run/climb stairs at 18 months (Centers for Disease Control and Prevention, 2017c). In our study, older children were more likely to fall from the same level or fall from stairs mechanisms. This finding is intuitive because older children have more independent mobility and may fall from running, tripping, or stumbling. Conversely, based on their dependent mobility, younger children were more likely to experience a multi-level fall because they were dropped from caregiver’s arms or inappropriately placed on household surfaces.

Similar to Unni et al.’s study (Unni et al., 2012), our study shows that a majority of ICD-9 CM.

E-codes in children < 1-year-old involve a MOI of multi-level fall including falling from caregiver’s arms, a bed, or furniture. In our study, children in this age group that fell from caregiver’s arms included those that were carried in baby carriers (such as car seats), by young children, or from caregiver’s falling asleep with patient in their arms. Gaw et al. (Gaw et al., 2017) reported that baby carriers were over five times more likely to be related to caregiver falls than other product groups and found a majority of their caregiver falls to be related to carrying child in a baby carrier. In our study, multiple cases of head injuries and extremity fractures resulted from children falling out of caregiver’s arms. Based on these findings, educational interventions should remind parents/caregivers to keep items off the floor and stairs to avoid tripping hazards, actively supervise younger children carrying infants/toddlers, and avoid using baby carriers (car seats, bouncy seats) to carry children outside of their intended purpose (in motor vehicles, stationary floor surfaces).

Like prior studies (Pomerantz et al., 2012; Kendrick et al., 2015; Khambalia et al., 2006; Pitone & Attia, 2006; Unni et al., 2012; Wang et al., 2013), our study found that many children fell from furniture beyond age 6 months. Educational interventions should remind parents/caregivers not to place infants on beds unsupervised since they can often roll or fall from a bed. Further, parents/caregivers should supervise toddlers and older children because they can climb onto and jump from beds/furniture and may push younger siblings from the bed. A majority, of the children that fell from a counter in our study were < 1-year-old and fell from a type of baby carrier (car seat, booster seat, bouncy seat) placed on top of the counter. Likewise, we also saw cases of children falling from baby carriers when they were placed on top of furniture. Kamboj et al. (Kamboj et al., 2017) reported infant patients were more likely than older children to sustain a traumatic brain injury from falling off, from, or with a product. Parents/caregivers should be advised to place all infant seats and carriers on the floor and not high surfaces. Current AAP anticipatory guideline recommendations review falls from furniture with parents/caregivers only through 6 months of age, falls from windows and stairs starting at 9 months (to continue through age 2), and outside play at 3-year-old and 4-year-old visits (Hagan et al., 2017). As reiterated by our data the current guidelines miss critical ages for continued supervision at home around furniture and should be expanded to address furniture falls beyond 6 months of age.

Although falls from playground and trampolines were mostly seen among older children in our study, there were cases identified among children < 1-year-old. To reduce playground injuries, parents/caregivers should increase supervision of young children on playground surfaces. Further, communities, daycare, and schools should routinely inspect and assess their playground surfaces and playground equipment to ensure they are updated, safe, and meet national consumer safety standards (American Society for Testing and Materials International, 2017). Trampolines should not be used for recreational use given inadequate standards for equipment safety and supervision from structured training programs (American Academy of Pediatrics, 2012).

Although infants have decreased mobility compared to older children, they are at higher risk of fall-related head injuries (even from low heights) because of their larger cephalic mass in proportion to the rest of their body (Wang et al., 2013). As a child ages, they have increased upper body strength, smaller head circumferences, and are able to brace their falls with their upper extremities (Kamboj et al., 2017). In our study, these anatomical characteristics are reflected by the higher proportion of head injuries among younger children study and the higher proportion of upper extremity injuries among older children.

We found a statistically significant younger Medicaid population over private payer presenting with falls. Black patients were also significantly younger in our dataset and had been found to be more likely on Medicaid. Often these families with public insurance seek the ED for minor illnesses outside of their acute injury visits (Flores & Tomany-Korman, 2008). These caregivers also may not be able to leave their jobs during the day due to sole income and other hardships and thus utilize the ED after hours for minor illnesses. It is in these instances where brief targeted age appropriate falls education can be given. Providing age-appropriate and brief injury prevention education in an ED setting at non-urgent visits may add to current public health efforts in reducing pediatric falls. Another potential option for injury prevention education in this demographic would be to provide falls education at local governmental resource offices such as Women, Infants, and Children (WIC).

The majority of our patients were admitted to the general inpatient floor, which reinforces previous research evaluating trauma registry data for falls among children < 5 years old (Pomerantz et al., 2012). Reasons for admission included medical care of injuries, observation of children < 1-year-old with head injuries, concerns for possibility of child abuse, and parental request for admission. Children that were admitted to the operating room were older relative to those admitted to the ICU. This correlates with the higher percentage of orthopedic extremity fractures needing surgical repair among older children compared to the higher percentage of non-operatively managed head injuries among younger children in the ICU. The majority of our patient population were transferred from an outside facility and accounted for most of the injuries seen, admissions, and discharges. One prior study found that EDs with a larger proportion of Medicaid patients had a higher odds of transferring patients with Medicaid over private payer (Huang et al., 2017). Of the remaining 30% of children in the GCTR database that were not transferred to CHOA for care, the majority received care at either an adult level 1 or level 2 trauma center.

### Limitations

There were limitations in our study. First, despite efforts by the trauma registrar coordinators and investigators to identify abuse (review medical records including social work, ED, child advocacy, hospital summary and discharge notes), it is possible the research team was unable to identify and exclude all intentional injury cases based on available data. Second, our data only included patients in the trauma registry, which represents the most severe cases and explains our high admission rate in our dataset. Thus, our results may not be generalizable to other institutions that primarily see non-acute falls. Third, the large percentages of ‘other’ or ‘unknown’ MOI and ‘unknown’ height should be considered when interpreting the results of this study. Fourth, since this retrospective study used ICD-9 coding, patient misclassification could have occurred and led to under- or over-reporting. Similarly, investigators used product coding based on medical record review for specific mechanisms, which could have been misclassified leading to under- or over-counting reporting. Additionally, MOI is reliant on caregiver reports, which could have been inaccurate in some instances. Fifth, ISS is based on the three most severely injured body regions and does not account for multiple severe injuries within the same body region (Smith et al., 2015). This limitation of the ISS can result in a misleadingly low or high score based on which injuries are included in the scoring system.

## Conclusions

Despite being highly preventable, unintentional pediatric falls are the leading cause for childhood injury ages 0–4 years. Our research identified trends for children < 1-year-old having a majority of low-level falls and sustaining majority of severe head injuries. Children < 1-year-old most frequently experienced multi-level falls MOI predominantly from bed or caregiver’s arms while older children primarily experienced multi-level falls MOI from furniture and outdoor surfaces. In comparison to older children, those < 1-year-old had more household falls from baby carriers being placed on raised surfaces. Our study showed larger proportions of younger Medicaid patients sustaining falls.

Further prevention efforts should target low height falls at home with education from pediatric clinics, emergency departments, and community centers/daycares. Health care providers and community workers should consider age-appropriate recommendations and population-based targeted education towards caregivers using injury and demographic patterns identified in this study. Parents/caregivers should be advised on recommended best practices for supervision and care of young children both indoors and outdoors to prevent falls effectively. They should be given education on available products and behaviors to reduce falls including: 1) use of window guards and stair gates; 2) avoidance of placement of children on high surfaces both with and without baby carriers; 3) only placing baby carriers on ground surfaces; 5) use of safety belts with baby carriers, high chairs, and changing tables; 6) safe play recommendations for playgrounds and bounce houses; and 7) avoidance of use of trampolines for child recreational play.

## Additional file

Additional file 1: Tables S1-Tables S3. This additional file contains the Appendix for the manuscript. Table S1 lists all of the ICD-9-CM E-codes used for the study population. Table S2 lists product coding performed by investigators. Table S3 shows the post hoc comparisons for Table 2. (PDF 101 kb)

## Abbreviations

AAP: American Academy of Pediatrics
AIS: Abbreviated Injury Score
CHOA: Children’s Healthcare of Atlanta
CI: Confidence interval
COIVPP: Council on Injury, Violence, and Poison Prevention
E-code: External cause of injury code
ED: Emergency department
GCTR: Georgia Central Trauma Registry
ICD-9-CM: International classification of diseases, ninth revision, clinical modification
ICU: Intensive care unit
ISS: Injury Severity Score
LOS: Length of stay
MOI: Mechanism of injury
MRN: Medical record number
NTDSD: National Trauma Data Standard Dataset
OR: Odds ratio
SES: Socioeconomic status
WIC: Women Infant and Children
