# Supplementary material for: Pediatric falls ages 0–4: understanding demographics, mechanisms, and injury severities
Source: Inj Epidemiol. 2018 Apr 10;5(Suppl 1):7. doi: 10.1186/s40621-018-0147-x (PMC5893510; doi:10.1186/s40621-018-0147-x)
Supplement: Supplementary file 1 — Tables S1-Tables S3. This additional file contains the Appendix for the manuscript. Table S1 lists all of the ICD-9-CM E-codes used for the study population. Table S2 lists product coding performed by investigators. Table S3 shows the post hoc comparisons for Table 2. (PDF 101 kb) [file 40621_2018_147_MOESM1_ESM.pdf]

Appendix

Table A1. ICD9-CM Unintentional Fall E-codes

ICD9 CM Fall Mechanism of Injury E codes
880.1
**Fall On or From Stairs/Steps - Sidewalk Curb, Not Applicable**
880.9
**Fall On or From Stairs/Steps - Oth Stairs or Steps**
**Fall On or From Stairs/Steps - Oth Stairs or Steps, Not Applicable**
**Fall On or From Stairs/Steps - Oth Stairs or Steps, Oth/Unspec Injury, Un/Intentional - Oth Spec Means**
**Fall On or From Stairs/Steps - Oth Stairs or Steps, Striking Against/Struck Accidentally - Oth Stationary Object w/o Subseq Fall**
**Fall On or From Stairs/Steps - Oth Stairs or Steps, Striking Against/Struck Accidentally - Oth w/ or w/o Subseq Fall**
**Fall On or From Stairs/Steps - Oth Stairs or Steps, Not Applicable**
882
**Fall From or Out of Building/Other Structure**
**Fall From or Out of Building/Other Structure, Not Applicable**
883.2
**Fall into Hole/Oth Surface Opening - Storm Drain/Manhole, Not Applicable**
883.9
**Fall into Hole/Oth Surface Opening - Oth Hole/Surface Opening**
**Fall into Hole/Oth Surface Opening - Oth Hole/Surface Opening, Not Applicable**
884
**Oth Multi-level Fall - Playground Equipment**
**Oth Multi-level Fall - Playground Equipment, Not Applicable**
**Oth Multi-level Fall - Playground Equipment, Oth/Unspec Injury, Un/Intentional - Oth Spec Means**
884.2
**Oth Multi-level Fall - Chair**
**Oth Multi-level Fall - Chair, Not Applicable**
884.4
**Oth Multi-level Fall - Bed**
**Oth Multi-level Fall - Bed, Not Applicable**
**Oth Multi-level Fall - Bed, Oth/Unspec Injury, Un/Intentional - Unspec Means**
**Oth Multi-level Fall - Bed, Striking Against/Struck Accidentally - Furniture w/ Subseq Fall**

**Oth Multi-level Fall - Bed, Striking Against/Struck Accidentally - Oth Stationary Object w/ Subseq Fall**
**Oth Multi-level Fall - Bed, Unknown**
**Oth Multi-level Fall - Bed, Not Applicable**
884.5
**Oth Multi-level Fall - Other Furniture**
**Oth Multi-level Fall - Other Furniture, Not Applicable**
**Oth Multi-level Fall - Other Furniture, Oth Multi-level Fall - Chair**
**Oth Multi-level Fall - Other Furniture, Striking Against/Struck Accidentally - Oth Stationary Object w/o Subseq Fall**
**Oth Multi-level Fall - Other Furniture, Striking Against/Struck Accidentally - Oth w/ or w/o Subseq Fall**
**Oth Multi-level Fall - Other Furniture, Unknown**
884.9
**Oth Multi-level Fall - Oth Multi-Level Fall**
**Oth Multi-level Fall - Oth Multi-Level Fall, Fall On or From Stairs/Steps - Oth Stairs or Steps**
**Oth Multi-level Fall - Oth Multi-Level Fall, N-traffic Accident, Oth Off-Road MV - Passenger in MV, Non MC**
**Oth Multi-level Fall - Oth Multi-Level Fall, Not Applicable**
**Oth Multi-level Fall - Oth Multi-Level Fall, Oth and Unspec Fall - Resulting in Striking Other Object**
**Oth Multi-level Fall - Oth Multi-Level Fall, Oth Multi-level Fall - Playground Equipment**
**Oth Multi-level Fall - Oth Multi-Level Fall, Oth/Unspec Injury, Un/Intentional - Unspec Means**
**Oth Multi-level Fall - Oth Multi-Level Fall, Striking Against/Struck Accidentally - Oth Stationary Object w/ Subseq Fall**
**Oth Multi-level Fall - Oth Multi-Level Fall, Striking Against/Struck Accidentally - Oth Stationary Object w/o Subseq Fall**
**Oth Multi-level Fall - Oth Multi-Level Fall, Struck Accidentally by Falling Object**
**Oth Multi-level Fall - Oth Multi-Level Fall, Unknown**
**Oth Multi-level Fall - Oth Multi-Level Fall, Not Applicable**
885
**Fall on Same Level - Nonmotorized Scooter (10/2002)**
**Fall on Same Level - Nonmotorized Scooter (10/2002), Not Applicable**
885.9
**Fall on Same Level - Other**
**Fall on Same Level - Other, Caught Accidentally In or Between Objects**
**Fall on Same Level - Other, Cutting Object Accident - Oth Spec Cut/Piercing Instrument/Object**

**Fall on Same Level - Other, Cutting Object Accident - Unspec Cut/Piercing Instrument/Object**
**Fall on Same Level - Other, Foreign Body Accidentally Entering Oth Orifice**
**Fall on Same Level - Other, Not Applicable**
**Fall on Same Level - Other, Oth and Unspec Fall - Resulting in Striking Other Object**
**Fall on Same Level - Other, Oth Multi-level Fall - Bed**
**Fall on Same Level - Other, Striking Against/Struck Accidentally - Furniture w/ Subseq Fall**
**Fall on Same Level - Other, Striking Against/Struck Accidentally - In Sports w/o Subseq Fall**
**Fall on Same Level - Other, Striking Against/Struck Accidentally - Oth Stationary Object w/ Subseq Fall**
**Fall on Same Level - Other, Striking Against/Struck Accidentally - Oth Stationary Object w/o Subseq Fall**
**Fall on Same Level - Other, Striking Against/Struck Accidentally - Oth w/ or w/o Subseq Fall**
**Fall on Same Level - Other, Striking Against/Struck Accidentally - Oth Stationary Object w/o Subseq Fall**
**Fall on Same Level - Other, Cutting Object Accident - Oth Spec Cut/Piercing Instrument/Object**
**Fall on Same Level - Other, Not Applicable**
886.9
**Fall From Collision/Push/Shoving By, W/ Oth Person - Oth/Unspec**
**Fall From Collision/Push/Shoving By, W/ Oth Person - Oth/Unspec, Not Applicable**
**Fall From Collision/Push/Shoving By, W/ Oth Person - Oth/Unspec, Striking Against/Struck Accidentally - Oth w/ or w/o Subseq Fall**
**Fall From Collision/Push/Shoving By, W/ Oth Person - Oth/Unspec, Not Applicable**
**Fall From Collision/Push/Shoving By, W/ Oth Person - Oth/Unspec, Not Applicable**
888
**Oth and Unspec Fall - Resulting in Striking Sharp Object**
**Oth and Unspec Fall - Resulting in Striking Sharp Object, Cutting Object Accident - Oth Spec Cut/Piercing Instrument/Object**
888.1
**Oth and Unspec Fall - Resulting in Striking Other Object**
**Oth and Unspec Fall - Resulting in Striking Other Object, Not Applicable**
888.8
**Oth and Unspec Fall - Oth**
888.9
**Oth and Unspec Fall - Unspec**
**Oth and Unspec Fall - Unspec, Not Applicable**
**Oth and Unspec Fall - Unspec**
920.8

**Cutting Object Accident - Oth Spec Cut/Piercing Instrument/Object, Fall on Same Level - Other**
**Cutting Object Accident - Oth Spec Cut/Piercing Instrument/Object, Oth and Unspec Fall - Resulting in Striking Sharp Object**
**Cutting Object Accident - Oth Spec Cut/Piercing Instrument/Object, Oth Multi-level Fall - Bed**
988.9
**Oth/Unspec Injury, Un/Intentional - Unspec Means, Oth Multi-level Fall - Bed**

Table A2. Product Coding for Fall Injury

Product Category*	Definition-multiple
**Chair**	chair, ottoman, barstool, stool, bench
**Bed**	bed, bunk bed
**Playground**	playground (not otherwise specified), monkey bars, swing, slide, merry go round, jungle gym, see saw, stairs on playground equipment
**Table**	table, coffee table
**Other Furniture**	furniture (not otherwise specified), desk, dresser, indoor swing, TV stand, nightstand
**Vehicle**	car, truck, tractor trailer, golf cart
**Toy**	toy (not otherwise specified), rocking horse, play kitchen
**Wheeled toy**	bicycle, tricycle, small ride, non-motorized scooter, wagon, power wheel

*The following product categories have single definitions: Counter, bouncy house, bouncy seat, booster seat, changing table, crib, couch, shopping cart, stroller, high chair, trampoline

Table A3. Post-hoc comparisons (Mann-Whitney U tests) for Table 2

Post-hoc Comparisons for Payor

Comparison	p
Medicaid vs Private	<.001
Medicaid vs Other	.001
Private vs Other	.61

Post-hoc Comparisons for Race

Comparison	p
Black vs White	<.001
Black vs Other	.001
White vs Other	.65

Post-hoc Comparisons for ED Disposition

Comparison	p
ICU vs Bed	.48
ICU vs Home	.17
ICU vs OR	.01
Bed vs Home	.16
Bed vs OR	.003
Home vs OR	.06

Post-hoc for Comparisons for General MoI

Comparison	p
Fall on/from Stairs vs Multilevel Fall	.27
Fall on/from Stairs vs Other	.001
Fall on/from Stairs vs Fall on the Same Level	<.001
Multilevel Fall vs Other	.001
Other vs Fall on the Same Level	.53

Post-hoc Comparisons for ICD-9 MoI (Specific)

Comparison	p
Bed vs Stairs	.13
Bed vs Furniture	.03
Bed vs Other	<.001
Bed vs Playground Equipment	<.001
Stairs vs Furniture	.76
Stairs vs Other	.01
Stairs vs Playground Equipment	<.001
Furniture vs Other	.01
Furniture vs Playground Equipment	<.001
Other vs Playground Equipment	<.001

Post-hoc for ISS Level

Comparison	p
1-8 vs 9-15	<.001
1-8 vs 16-25	<.001
9-15 vs 16-25	.002
